# Ectopic Expression of *CsCTR1*, a Cucumber CTR-Like Gene, Attenuates Constitutive Ethylene Signaling in an *Arabidopsis ctr1-1* Mutant and Expression Pattern Analysis of *CsCTR1* in Cucumber (*Cucumis sativus*)

**DOI:** 10.3390/ijms150916331

**Published:** 2014-09-15

**Authors:** Beibei Bie, Jin Sun, Junsong Pan, Huanle He, Run Cai

**Affiliations:** 1 Plant Science Department, School of Agriculture and Biology, Shanghai Jiaotong University, Shanghai 200240, China; E-Mails: biepeipei@hotmail.com; (B.B.); jspan71@sjtu.edu.cn (J.P.); hlhe75@sjtu.edu.cn (H.H.); 2 National-Local Joint Engineering Research Center of Biodiagnostics & Biotherapy, Xi’an Jiaotong University, Xi’an 710004, Shaanxi, China; E-Mail: jinsun2012@outlook.com

**Keywords:** *Cucumis sativus*, *constitutive triple response 1* (*CTR1*), ectopic expression, ethylene signaling, expression analysis

## Abstract

The gaseous plant hormone ethylene regulates many aspects of plant growth, development and responses to the environment. *Constitutive triple response 1* (*CTR1*) is a central regulator involved in the ethylene signal transduction pathway. To obtain a better understanding of this particular pathway in cucumber, the cDNA-encoding *CTR1* (designated *CsCTR1*) was isolated from cucumber. A sequence alignment and phylogenetic analyses revealed that CsCTR1 has a high degree of homology with other plant CTR1 proteins. The ectopic expression of *CsCTR1* in the *Arabidopsis ctr1-1* mutant attenuates constitutive ethylene signaling of this mutant, suggesting that CsCTR1 indeed performs its function as negative regulator of the ethylene signaling pathway. *CsCTR1* is constitutively expressed in all of the examined cucumber organs, including roots, stems, leaves, shoot apices, mature male and female flowers, as well as young fruits. *CsCTR1* expression gradually declined during male flower development and increased during female flower development. Additionally, our results indicate that *CsCTR1* can be induced in the roots, leaves and shoot apices by external ethylene. In conclusion, this study provides a basis for further studies on the role of CTR1 in the biological processes of cucumber and on the molecular mechanism of the cucumber ethylene signaling pathway.

## 1. Introduction

Ethylene (C_2_H_4_) was the first example of a gaseous signaling molecule involved in biological systems and was discovered more than a century ago. Ethylene regulates a variety of developmental and stress responses in plants, including seed germination, cell elongation, cell fate, sex determination, fruit ripening, flower senescence, leaf abscission, defense against pathogens, and responses to mechanical trauma and cold and salt stresses [1]. Ethylene-regulated processes can be controlled at the level of hormone biosynthesis and perception and the signal transduction pathway. Components of the ethylene signal transduction pathway were identified using *Arabidopsis* mutants that were altered in the seedling triple response [2], and a basic model of the ethylene signal transduction pathway has been established [3].

Ethylene is perceived in *Arabidopsis* by a family of receptors (ETR1, ETR2, ERS1, ERS2 and EIN4) similar to the bacterial two-component histidine kinase sensors [4,5,6]. These receptors are localised in the Endoplasmic reticulum (ER) membrane and Golgi apparatus [7]. Studies have found that all of these genes have redundant functions as negative regulators of ethylene signaling, and their double, triple and quadruple mutants result in constitutive responses to ethylene [5,8].

Acting downstream of the receptors is a Raf-like serine/threonine protein kinase *constitutive triple response 1* (*CTR1*), a central component in the ethylene signal transduction pathway and the first gene in the ethylene signal transduction pathway to be cloned in *Arabidopsis* [9]. CTR1 is a negative regulator that actively suppresses the ethylene signal response in the absence of ethylene. After the binding of ethylene to the receptors, CTR1 becomes inactivated, and the ethylene-responsive pathway is triggered via other downstream components, including *EIN2* and *EIN3/EIL* [9,10]. CTR1 consists of a unique *N*-terminal regulatory domain and a *C*-terminal serine/threonine kinase domain. The *N*-terminal domain of CTR1 shares little sequence homology with the *N*-terminus of Raf protein-serine/threonine kinase homologues and has been shown to interact with the histidine kinase domain of ethylene receptors and to be important in the negative regulation of ethylene signaling by CTR1. In contrast, the *C*-terminal domain of CTR1 exhibits a high degree of homology with the Raf kinase in mammals [8,11]. Loss-of-function mutations in the *CTR1* gene lead to a triple-response phenotype in the absence of exogenous ethylene and the constitutive expression of ethylene-regulated genes, indicating that the *CTR1* gene product acts as a key negative regulator in the ethylene signal transduction pathway [2,9]. CTR1 can interact with and directly phosphorylates the cytosolic *C*-terminal domain of ethylene-insensitive 2 (EIN2), an ER membrane-localised Nramp homolog that positively regulates ethylene responses. The phosphorylation of EIN2 by CTR1 prevents EIN2 from signaling in the absence of ethylene, whereas the inhibition of CTR1 upon ethylene perception results in the cleavage of EIN2 and the movement of the EIN2 *C*-terminus into the nucleus, allowing the ethylene signal to reach and activate downstream transcription factors [12,13,14,15]. A recent study found that CTR1 activation by ethylene receptors might require ECR2 (enhancing ctr1-10 ethylene response2) for suppressing the ethylene response. ECR2 is a novel allele involved in the ethylene receptor signaling that is mediated by CTR1 [16].

Interestingly, the *CTR1* gene is unique in the *Arabidopsis* genome [9]. However, in tomato it belongs to a multigenic family including four *CTR*-like genes (*LeCTR1*, *LeCTR2*, *LeCTR3* and *LeCTR4*) [17,18,19], among which LeCTR1, LeCTR3 and LeCTR4 are highly similar to AtCTR1, and LeCTR2 is similar to AtEDR1 [17], and each of the genes appears to be involved in different tissues and processes. *LeCTR2* is constitutively expressed and unresponsive to ethylene [20]; *LeCTR1* is up-regulated by ethylene and increases during fruit ripening [17,18]; *LeCTR3* and *LeCTR4* exhibits a higher level of expression in leaves than in fruits and do not respond to ethylene [17]. In *Cucurbita pepo*, two *CTR*-like genes (*CpCTR1* and *CpCTR2*) have been cloned and characterised. The transcripts of both of these genes were detected in different plants and organs, including roots, leaves and shoots, but mostly accumulated in mature flowers [21]. Additionally, in the earlier stages of flower development, the expression of *CpCTR1* and *CpCTR2* is higher in male floral organs [21]. Two *CTR*-like genes have also been identified in cut rose and kiwifruit, and these two *CTR*-like genes also exhibited different expression patterns [22,23]. In contrast, in the genomes of other species, such as pear [24], delphinium [25], dplum [26] and apple [27], a *CTR1*-like gene appears to be unique as in the *Arabidopsis* genome.

Cucumber is an economically important crop as well as a model system for sex determination studies due to its rich diversity of floral sex types. Ethylene has been demonstrated to promote female flower development and is highly correlated with femaleness in cucumber [28]. Interestingly, gynoecious lines of cucumber generate more ethylene than do monoecious or andromonoecious line [29,30]. To date, several genes that determine the ratio of male-to-female flowers in cucumber have been isolated and characterised. All of these genes encode 1-aminocyclopropane-1-carboxylic acid synthase (ACS), the key rate-limiting enzyme for ethylene biosynthesis in higher plants [31,32]. Compared with the ethylene biosynthesis pathway, very little is known about the role of ethylene signaling in the sex determination of flowers, mainly because the key components for the ethylene signaling pathway have not yet been identified in cucumber. So far, only a few of these components have been cloned from cucumber, including ETR2, ETR1, ERS and EIN3 [33,34]. Therefore, the majority of the key components remain to be isolated and characterized.

Given that the *CTR1* genes are key negative regulators in the ethylene signal transduction pathway, they may play an important role in multiple biological processes in cucumber, especially in sex determination and flower development. However, there is very little information about the *CTR1* genes in cucumber. It was first necessary to isolate and characterise the *CTR1* genes from cucumber before addressing its function.

In the present study, a cDNA clone of the cucumber *CTR1*-like gene, *CsCTR1*, was cloned through a search of tentative consensus and UniGene sequences in the cucumber genome database and through a bioinformatics analysis. To investigate the functional role of CsCTR1, we ectopically expressed *CsCTR1* in an *Arabidopsis ctr1-1* mutant. Furthermore, we also examined the expression pattern of *CsCTR1* in various cucumber organs and in different developmental stages of both male and female flowers, as well as this gene’s response to external ethylene treatment. The data that are presented in this paper lay the groundwork for further studies of *CTR1* functions in cucumber.

## 2. Results

### 2.1. Cloning of Open Reading Frame (ORF) of CsCTR1 and Sequence Analysis

**Figure 1:**
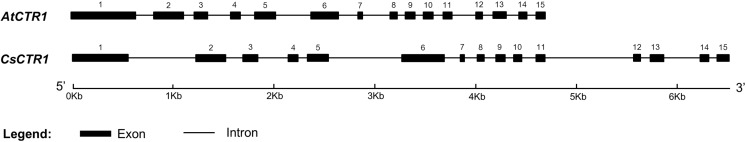
Comparison of the genomic structure of cucumber *CsCTR1* (JQ277220) and the *Arabidopsis AtCTR1* gene (L08790). Black portions represent the exons, straight lines represent the introns.

A cDNA clone of *CsCTR1* (GenBank accession no. JQ277220) from cucumber was cloned based on the tentative consensus sequence (csa011978). A sequence analysis showed that the open reading frame (ORF) of *CsCTR1* was 2559 bp in length, encoding a putative polypeptide of 852 amino acids with a molecular mass of 94.521 kDa and isoelectric point of 5.77. The BLASTN search result, using the nucleotide sequence of *CsCTR1* against the chromosome database within the cucumber genome database, showed that *CsCTR1* was a single copy gene in the cucumber genome. A gene structure analysis using GSDS (gene structure display server) tools indicated that the *CsCTR1* genomic DNA sequence that was obtained from the cucumber genome database contained 15 exons and 14 introns, similar to the *Arabidopsis CTR1* (*AtCTR1*), and the position and size of the exons in *CsCTR1* exhibited a high degree of similarity with those in *AtCTR1* (Figure 1). In addition, two putative ethylene-responsive elements (ERE, A(A/T)TTCAAA) were found in the *CsCTR1* promoter and were located 490 and 1308 bp upstream of the ATG codon when using Plant-CARE [35] to identify the *cis*-acting regulatory element in the promoter sequence. It is implied that the transcription of *CsCTR1* might be induced by ethylene.

### 2.2. Multiple Sequence Alignment and Phylogenetic Analysis

**Figure 2:**
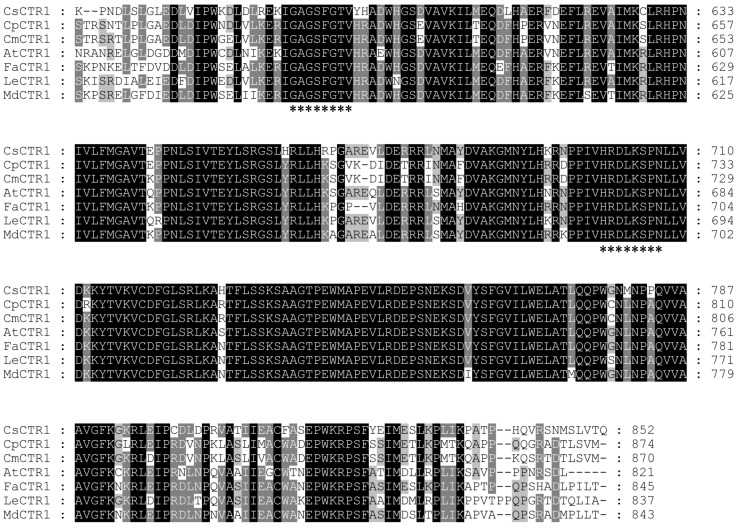
Multiple amino acid sequence alignments of the *C*-terminal Ser/Thr kinase domain in the CTR1-like proteins of several plant species. Seven genes from *Malus* × *domestica* (MdCTR1, ABI58290), *Solanum lycopersicum* (LeCTR1, AAL87456), *Cucumis melo* (CmCTR1, ADV92636), *Cucurbita pepo* (CpCTR1, ADB55631), *Arabidopsis thaliana* (AtCTR1, CAB82938), *Fragaria* × *ananassa* (FaCTR1, AFI38955) and *Cucumis sativus* (CsCTR1, AEZ53932) were used for multiple sequence alignment. Amino acids with identities of over 75% are shaded in black; whereas, those with identities ranging between 50% and 75% are shaded in gray. The ATP binding signature (GxGxxGxV) and the protein kinase active site consensus sequence (HRDLKxxN) are indicated by asterisk. Numbers for amino acids are indicated on the right.

Multiple amino acid sequence alignments revealed that the molecular structure of *CTR1*-like proteins appears to be highly conserved in different species. These sequences correspond to proteins of approximately 800 amino acids with a conserved *C*-terminal serine/threonine protein kinase domain (Figure 2). CsCTR1 had a very high degree of homology with the counterparts of other plant species; CsCTR1 has identities of 66%, 65%, 65%, 65%, 63% and 63% with CmCTR1, CpCTR1, MdCTR1, FaCTR1, LeCTR1 and AtCTR1, respectively. We found that the *C*-terminal end of CsCTR1 had a highly conserved serine/threonine protein kinase domain, which is also present in all of the CTR1-like proteins. The ATP binding site (GAGSFGTV) and the protein kinase active site (HRDLKSPN) [36] were also found in the serine/threonine protein kinase domain of CsCTR1 (Figure 2).

**Figure 3:**
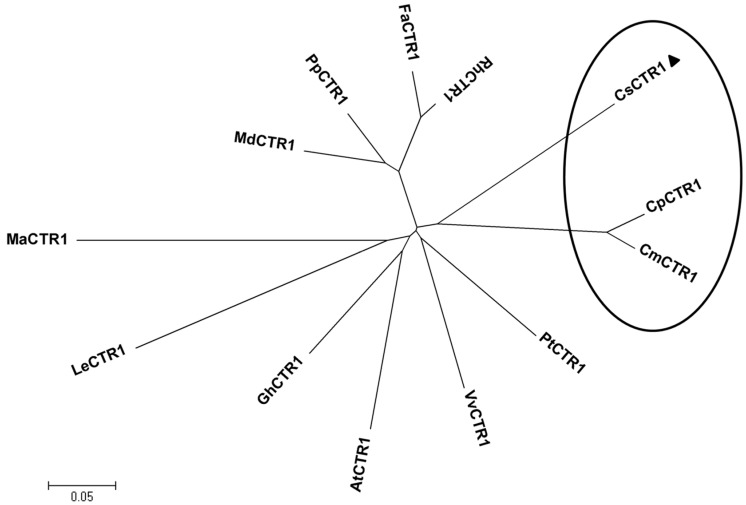
Phylogenetic relationships of known CTR1 in plants. Amino acid sequences used for alignment include *Cucumis melo* (CmCTR1, ADV92636), *Vitis vinifera* (VvCTR1, XP_002277360), *Cucurbita pepo* (CpCTR1, ADB55631), *Prunus persica* (PpCTR1, ACR23642), *Malus* × *domestica* (MdCTR1, ABI58290), *Rosa hybrid cultivar* (RhCTR1, AAK40361), *Fragaria* × *ananassa* (FaCTR1, AFI38955), *Gossypium hirsutum* (GhCTR1, ACZ66010), *Populus trichocarpa* (PtCTR1, XP_002326245), *Solanum lycopersicum* (LeCTR1, AAL87456), *Arabidopsis thaliana* (AtCTR1, CAB82938), *Musa acuminata* (MaCTR1, AFA37962) and *Cucumis sativus* (CsCTR1, AEZ53932). The scale bar represents the estimated evolutionary distance as 0.05 amino acid substitutions per site. CsCTR1 (indicated with black triangles) is grouped together with counterparts from melon (CmCTR1) and pumpkin (CpCTR1), as indicated by the ellipse.

To understand the evolutionary relationships among CsCTR1 and other plant CTR1 proteins, a phylogenetic tree was constructed based on the amino acid sequences of another 12 plant CTR1 genes. The plant CTR1 proteins were derived from a common ancestor during evolution, and CsCTR1 was grouped into a cluster with the CTR1 proteins from melon and pumpkin, which belong to the Cucurbitaceae (Figure 3).

### 2.3. Ectopic Expression of CsCTR1 in an Arabidopsis ctr1-1 Mutant

**Figure 4:**
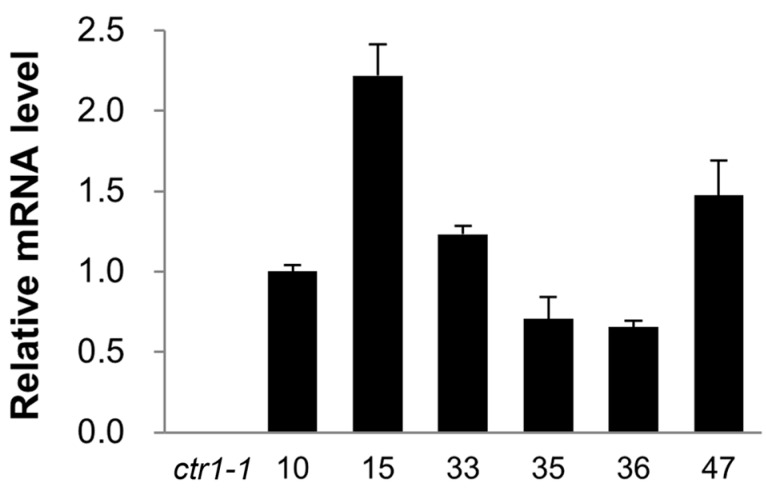
Expression analysis of *CsCTR1* in *Arabidopsis* transgenic line 10, 15, 33, 35, 36, 47 and *ctr1-1* mutant by quantitative real-time PCR. Each result was the average of three independent biological replicates. Error bars on each column indicate standard deviation from three replicates.

To assess the functional significance of *CsCTR1*, we conducted a complementation assay of the *Arabidopsis ctr1-1* mutant (Columbia ecotype) using an expression vector containing the *CsCTR1* cDNA driven by the 35S promoter. We obtained six homozygous transgenic lines by *A. tumefaciens*-mediated transformation, lines 10, 15, 33, 35, 36 and 47. Kanamycin resistance screening of the harvested T1 seeds demonstrated that all six of these transgenic lines were a result of single-copy integration according to their segregation ratio of 3:1. An expression analysis using qRT-PCR demonstrated that the transcripts of *CsCTR1* could be detected in all six of the transgenic lines and that lines 15, 33 and 47 exhibited greater expression than the other ones (Figure 4).

### 2.4. Partial Complementation of the Arabidopsis ctr1-1 Mutant by CsCTR1

**Figure 5:**
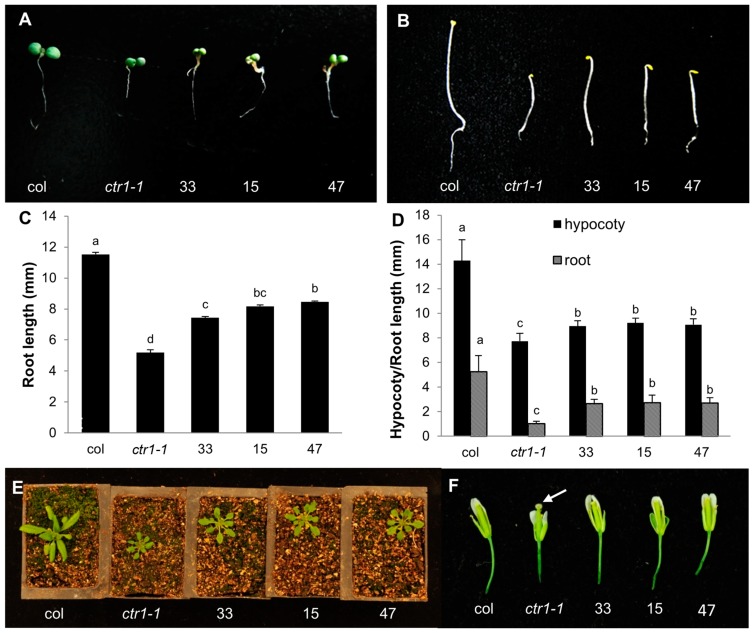
Phenotypes of the transgenic *CsCTR1*-overexpressing lines (33, 15 and 47) compared with that of *Arabidopsis* wild type (col) and the *ctr1-1* mutant. (**A**) Five-day-old seedlings grown in the light; (**B**) Five-day-old etiolated seedlings; (**C**) Root length of the seedlings grown in the light; (**D**) Root and hypocotyl length of the etiolated seedlings; (**E**) Adult plants at the rosette stage; and (**F**) Phenotypes of the gynoecium in developing flower, the white arrow indicates the gynoecium protruding out of the unopened buds in *ctr1-1* mutant. Error bars on each column indicate standard deviation from ten replicates. The statistical significance was determined by Duncan’s multiple comparison tests. Different letters above bars indicate significant differences (*p* < 0.05). The same letter indicates no significant difference (*p* < 0.05).

**Figure 6:**
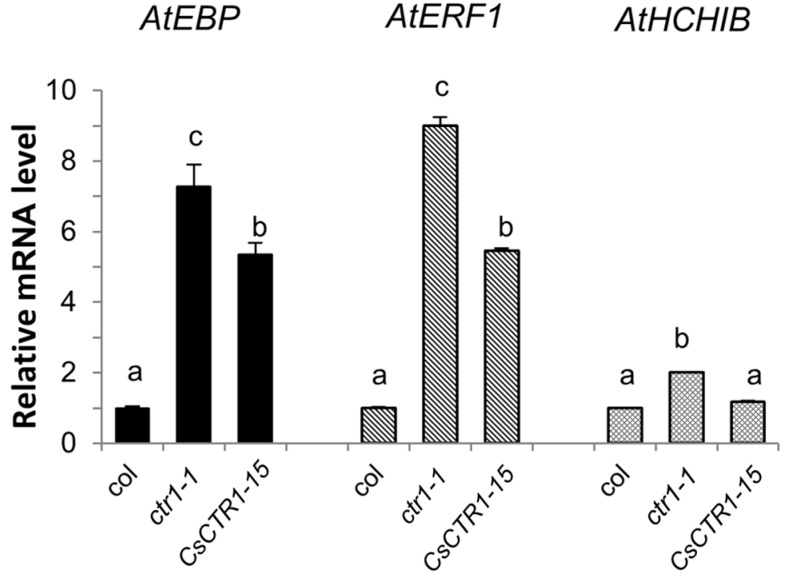
Expression analysis of *AtEBP*, *AtERF1* and *AtHCHIB* in *Arabidopsis* transgenic line 15. col: Columbia-0, *ctr1-1*: *Arabidopsis ctr1-1* mutant. *Arabidopsis Aactin 2* was used as internal control for qRT-PCR. Error bars on each column indicate standard deviation from three biological replicates. The statistical significance was determined by Duncan’s multiple comparison tests. Different letters above bars indicate significant differences (*p* < 0.05). The same letter indicates no significant difference (*p* < 0.05).

Kieber *et al.* (1993) found the *Arabidopsis ctr1-1* mutant plants showed a characteristic triple response phenotype, including short roots, short thickened hypocotyls and exaggerated apical hook, when grown in the air as well as in ethylene [9]. Adult plants of *ctr1-1* mutant had smaller rosette leaves and exhibited delayed flowering. Gynoecium of the mutants elongated significantly earlier relative to the rest of the developing flower, often protruding out of the unopened buds [9]. To evaluate the function of *CsCTR1* in the ethylene signaling pathway and the complementation capacity of *CsCTR1* in the *Arabidopsis ctr1-1* mutant, three transgenic lines, 15, 33 and 47, that had a high level of *CsCTR1* expression were examined for the ethylene-responsive phenotypes, specifically the lengths of the roots and hypocotyls, the size of the rosette leaves and the elongation of the gynoecium. We found that *CsCTR1* could partially restore the ethylene-responsive phenotype in the *ctr1-1* mutant. The root length of the light-grown transgenic seedlings was significantly longer than that of the *ctr1-1* mutant seedlings when measured 5 days after germination (Figure 5A,C; Table S1). The dark-grown etiolated transgenic seedlings also displayed a dramatic recovery of the hypocotyl and root elongation rate compared with that of the *ctr1-1* mutant (Figure 5B,D; Table S2). We also found that the size of the rosette leaves could be restored in transgenic lines to some extent (Figure 5E), and the gynoecium of the transgenic lines exhibited a similar phenotype to that of the wild type lines in that it did not protrude out of the unopened buds (Figure 5F). The transgenic lines flowered 5 to 7 days earlier than did the *ctr1-1* mutant, while col flowered 9 to 10 days earlier than did the *ctr1-1* mutant. Thus, it can be seen that the transgenic lines can partially restore this defect. We also examined the expression state of three ethylene-responsive genes, *AtEBP* (AT3G16770), *AtERF1* (AT3G23240) and *AtHCHIB* (AT3G12500), that were constitutively expressed in *ctr1-1* mutant. The qRT-PCR results demonstrate that the expression level of *AtEBP*, *AtERF1* and *AtHCHIB* significantly decreased in the transgenic lines and that the ectopic expression of *CsCTR1* could negatively regulate these ethylene-responsive genes (Figure 6).

### 2.5. Expression Patterns of CsCTR1 in Different Organs

**Figure 7:**
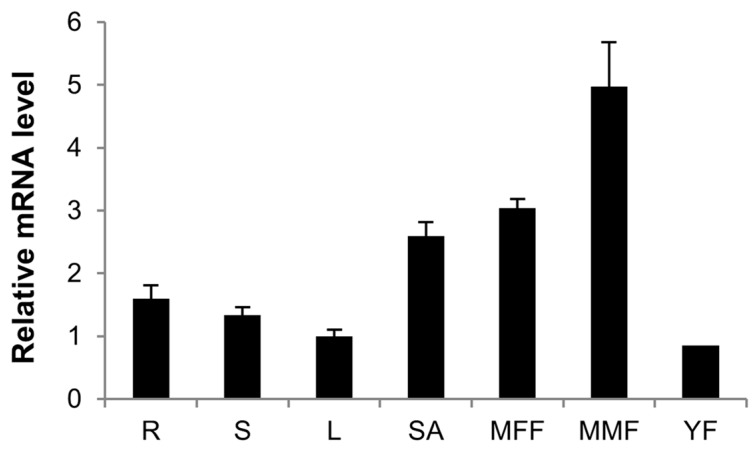
Expression patterns of *CsCTR1* in different organs of cucumber. Expression levels were assessed by qRT-PCR and were normalized to cucumber *Actin3*. Each result was the average of three independent biological replicates. Error bars on each point indicate standard deviation. R, roots; S, stem; L, leaves; SA, shoot apices; MMF, mature male flowers; MFF, mature female flowers; YF, young fruits.

To understand the role of *CsCTR1* in cucumber, we used qRT-PCR to determine the expression pattern of *CsCTR1* in different plant organs, including the roots, stems, leaves, shoot apices, and mature male and mature female flowers, as well as young fruits. We found that the highest level of *CsCTR1* expression was detected in mature male flowers, followed by mature female flowers and shoot apices (Figure 7). These results indicate that *CTR1*, a negative regulator of ethylene, facilitated the formation or development of male flowers and might simultaneously inhibit the female part of the same flower. Lower *CTR1* levels enhance ethylene signaling to aid in the formation of female flowers.

### 2.6. Expression Patterns of CsCTR1 during Flower Development

**Figure 8:**
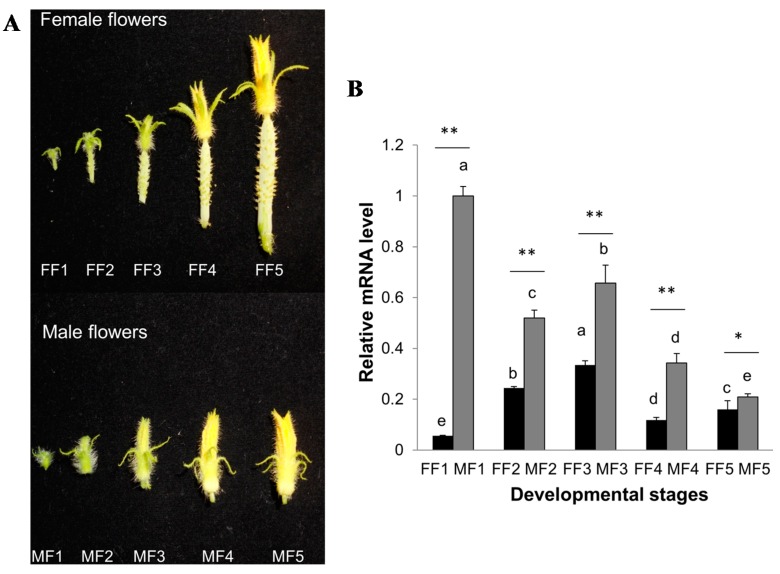
Expression patterns of *CsCTR1* during different flower developmental stages. (**A**) Morphology of male flowers (MF) and female flowers (FF) at five developmental stages, which were separated on the basis of the corolla length. MF1/FF1 = (2.5 ± 1) mm, MF2/FF2 = (5 ± 1) mm, MF3/FF3=(9 ± 2) mm, MF4/FF4=(15 ± 2) mm, MF5/FF5=(20 ± 2) mm; (**B**) Relative expression of *CsCTR1* normalized to the cucumber *Actin3* gene used as internal control in female flowers and male flowers at different developmental stages. Each result was the average of three independent biological replicates. Error bars on each column indicate standard deviation. The statistical significance was determined by Duncan’s multiple comparison tests and Student’s *t*-test. Different letters above bars indicate significant differences (*p* < 0.05). The same letter indicates no significant difference (*p* < 0.05). * *p* < 0.05, ** *p* < 0.01 by the Student’s *t*-test.

To understand the expression patterns of *CsCTR1* during flower development in cucumber, we explored the mRNA level of *CsCTR1* in five different developmental stages (MF1/FF1 to MF5/FF5) of female and male flowers. These floral developmental stages were separated on the basis of their corolla length and ranged from (2.5 ± 1) mm in MF1/FF1 to (20 ± 2) mm in MF5/FF5 floral buds (Figure 8A). We found that the accumulation of *CsCTR1* mRNA displayed a different trend between male and female flowers during development. Overall, the *CsCTR1* expression level in male flowers declined gradually with development, but in female flowers increased with development, although it declined slightly during stages FF4 and FF5 (Figure 8B). The discrepancy in the expression level of *CsCTR1* between male and female flowers was greatest during the FF1/MF1 stage and then narrowed gradually from the FF2/MF2 to FF5/MF5 stages. It is noteworthy that *CsCTR1* always exhibited a higher mRNA expression level in male flowers than that in female flowers during all five different floral developmental stages (Figure 8B).

### 2.7. Expression of CsCTR1 in Response to Ethylene and 1-Aminoethoxyvinylglycine (AVG) Treatment

**Figure 9:**
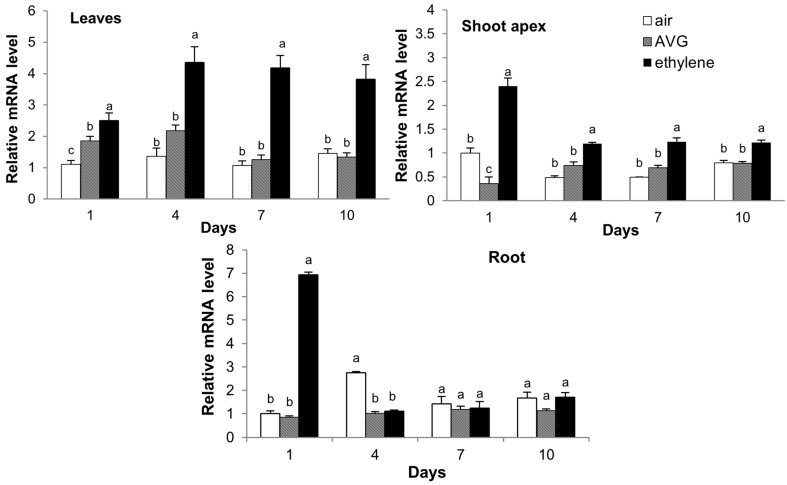
Expression of *CsCTR1* in response to ethylene and 1-aminoethoxyvinylglycine (AVG) treatment. The expression of *CsCTR1* in different organs was determined by qRT-PCR at 1, 4, 7 and 10 days after treatment with ethephon or AVG. Error bars on each column indicate standard deviation from three biological replicates. Columns with different lowercase letters are statistically different using the significant difference test of one-way ANOVA (*p*<0.05).

Previous studies have indicated that the expression of *CTR1* can be induced by endogenous or external ethylene [18,24,26,37]. Furthermore, we detected two putative ethylene-responsive elements (ERE) in the *CsCTR1* promoter region, implying that the expression of *CsCTR1* might be induced by ethylene. To assess the effects of the ethylene-releasing agent ethephon and ethylene synthesis inhibitor 1-aminoethoxyvinylglycine (AVG) on the expression of *CsCTR1*, we investigated the expression level of *CsCTR1* in the young leaves, shoot apices and roots that were collected 1, 4, 7 and 10 days after treatment. The expression of *CsCTR1* could be induced in all of the examined tissues, including the young leaves, shoot apices and roots. However, some differences in the induction situation existed among the three tissues (Figure 9). In leaves, the *CsCTR1* transcripts were significantly induced 1 day after ethephon treatment compared with the AVG and air treatments. The expression level continued to increase markedly 4 days after treatment and remained high until 10 days after treatment (Figure 9). In the shoot apices, the *CsCTR1* transcripts were significantly up-regulated by Ethephon 1 day after treatment. It is noteworthy that ethephon still affected the expression of *CsCTR1* even though the expression level dramatically decreased from 4 days after treatment (Figure 9). In the roots, interestingly, the expression of *CsCTR1* exhibited the greatest induction 1 day after ethephon treatment, and the inductive effect began to disappear from 4 days after treatment (Figure 9).

## 3. Discussion

CTR1, as key negative regulators of the ethylene signaling pathway, have still not been isolated from cucumber, thereby hampering the study on their biological functions in cucumber. In the present paper, we isolated a *CTR1-*like gene, *CsCTR1*, from the monoecious cucumber line S52. We found its genomic structural organisation was very conserved with that of other plant *CTR1* genes, such as *Arabidopsis AtCTR1* [9], tomato *LeCTR1* [18], and banana *MhCTR1* [37], including 15 exons and 14 introns. Such a high conservation of genomic structure might indicate a conserved function for these genes. However, we found the introns in *CsCTR1* were larger than those in *AtCTR1* despite their similar gene structure. The different intron sizes in different plant species could be due to different rates of non-coding sequence accumulation [17], which could provide an approach to differentiate among species.

A bioinformatics analysis showed that CsCTR1 possessed properties of the CTR1 protein by virtue of its highly conserved amino acid sequence when compared with that of other plant CTR1 genes. Particularly, CsCTR1 exhibited a high degree of homology with its counterpart CmCTR1 in melon and CpCTR1 in pumpkin and was grouped into the same cluster with melon and pumpkin, which belong to the same genus as cucumber. These results show that CsCTR1 might perform the same function in cucumber as do other plant CTR1 proteins.

We ectopically expressed *CsCTR1* in the *Arabidopsis ctr1-1* mutant to characterise the function of *CsCTR1*. The results demonstrate that the ectopic expression of *CsCTR1* in the *Arabidopsis ctr1-1* mutantcould partially attenuate constitutive ethylene signalling. We found that *CsCTR1* could partially restore the root and hypocotyl lengths, the size of the rosette leaves and the normal phenotype of the gynoecium in the *ctr1-1* mutant. Additionally, *CsCTR1* could down-regulate the ethylene-responsive genes that were constitutively expressed in the *ctr1-1* mutant. The complementation results indicate that *CsCTR1* indeed plays a critical role as a negative regulator of ethylene signalling. Here, we noticed that *CsCTR1* could not fully revert the constitutive ethylene signalling phenotypes of the *Arabidopsis ctr1-1* mutant. This may be explained by the fact that the expression of the 35S-driven *CsCTR1* cDNA could not be accurately regulated in the *Arabidopsis ctr1-1* mutant. Alternatively, this finding may be indicative of a functional divergence of CsCTR1 with respect to AtCTR1. Previous studies revealed that four *CTR*-like genes existed in the tomato genome and constitutive ethylene signalling phenotypes could not be reverted sufficiently by LeCTR1, whereas they could be reverted well by LeCTR3 and LeCTR4 when ectopically expressed in the *Arabidopsis ctr1-1* mutant via the CaMV 35S promoter [17]. This result indicates a likely functional divergence of CTR1 in cucumber. Using blast search, we found that another cucumber *CTR*-like gene (*csa005085*) was located on chromosome 3. Thus, we suspect that *csa005085* is likely to revert related phenotypes better than *CsCTR1*.

Previous studies have indicated that *CTR1* genes are expressed in almost all of the examined tissues, particularly in the reproductive organs such as flowers [18,21,37]. In this paper, we found the expression of *CsCTR1* could be detected in various organs, including the roots, stems, leaves, shoot apices, and mature male and female flowers, as well as young fruits. Consistent with previous studies [21], the expression level of *CsCTR1* was higher in the mature flowers, especially in male flowers. Furthermore, the transcript levels of *CsCTR1* were always higher in male flowers than in female flowers throughout floral development. Ethylene has been demonstrated to promote female flower development and is highly correlated with femaleness in cucumber. These results indicate that *CTR1*, a negative regulator of ethylene, facilitated the formation or development of male flowers and might simultaneously inhibit the female part of the same flower. Lower *CTR1* levels enhance ethylene signalling to aid in the formation of female flowers. Additionally, we also found that the difference in the *CsCTR1* expression level between male and female flowers was greatest during the FF1/MF1 stage, indicating that *CsCTR1* might play a critical role in early floral development.

The prediction of the *cis*-acting regulatory element found two putative ethylene-responsive elements (ERE) in the promoter region of *CsCTR1*; therefore, CTR1 might be regulated by ethylene. In this paper, our results demonstrate that the expression level of *CsCTR1* in all of the examined tissues, including the leaves, shoot apices and roots, could be induced upon exposure to external ethylene. Previous investigations have shown that the regulation of *CTR1-*like genes by ethylene differs between species. In *Arabidopsis*, *AtCTR1* is not inducible by ethylene in seedlings or adult plants [9,38]. However, for other plant species, such as tomato, pumpkin, rose, *Delphinium*, pear and plum, the expression of *CTR1* could be induced by ethylene in various tissues, including the roots, leaves, male flowers, female flowers and fruits [18,21,22,24,25,26,39]. Here, we also found that some differences in the inductive response of *CsCTR1* existed among the different tissues. The expression of *CsCTR1* in the leaves could be still induced by ethephon 10 days after treatment; as for root and shoot apices, the expression of *CsCTR1* began to decline 4 days after treatment. These results indicate that there might be some differences in the regulation of the response to ethylene among the different organs. However, all three detected tissues could be induced by exogenous ethylene. The phenomenon of ethylene increasing *CsCTR1* expression indicated that CsCTR1 might participate in the auto-inhibition of ethylene signaling pathway, similar to EBF2 (EIN3 binding F-Box2). Konishi and Yanagisawa (2008) found EBF2 can be induced by ethylene and involves feedback regulation of ethylene signaling by EIN3 in *Arabidopsis* [40].

In summary, CTR1 is a key negative regulator of the ethylene signal transduction pathway, playing important roles in the regulation of plant development. We isolated and characterised the *CTR1* gene from cucumber by its ectopic expression in an *Arabidopsis ctr1-1* mutant and investigated its expression patterns in different plant organs and during different stages of floral developmental, as well as in response to external ethylene. This study provides a foundation for further investigations into the role of CTR1 protein factors in the relevant biological processes of cucumber and the molecular mechanism of the cucumber ethylene signaling pathway.

## 4. Experimental Section

### 4.1. Plant Materials, Ethylene Treatments and Sampling

For cucumber, the monoecious plant line S52 (an inbred line derived from a southern Chinese local cultivar, Dabie Mountain, China) was used in all of the experiments and was provided by the Cucumber Research Group, School of Agriculture and Biology, Shanghai Jiaotong University, China. The plants were grown in the solid substrates (perlite:vermiculite:peat = 1:1:2). When the plants reached the 15- to 20-nodes stage, the taproot and branch root, main stems, mature leaves, shoot apices, young fruits, mature male flowers (MMF) and mature female flowers (MFF) on the day of anthesis, as well as both male and female flowers during five different developmental stages that could be distinguished based on the corolla length, were collected for the gene expression analysis. Regarding the flower samples, the entire flower body, including the calyx, ovary, stamen, pistil and petal, was collected. For the Ethephon (Sigma-Aldrich, Shanghai, China) and 1-aminoethoxyvinylglycine (AVG) (Sigma-Aldrich, Shanghai, China) treatments, the whole plants with four true leaves were sprayed with solutions containing either 0.1% (*v*/*v*) Tween 20 and 500 mg·L^−1^ ethephon or 0.1% (*v*/*v*) Tween 20 and 1 mM AVG. Meanwhile, the solid substrates were irrigated with solutions containing ethephon or AVG. The control plants were sprayed with only 0.1% (*v*/*v*) Tween 20. A total of 30 plants were used for each treatment. Young leaves, shoot apices and roots were collected for the gene expression analysis 1, 4, 7 and 10 days after treatment. All of the samples were frozen in liquid nitrogen after collection and stored at −80 °C until RNA extraction.

For *Arabidopsis thaliana*, Columbia-0 (Col-0) wild type and *ctr1-1* mutant (CS8057) plants were obtained from the *Arabidopsis* Biological Resource Centre (Columbus, OH, USA). The *Arabidopsis* plants were grown in a plant growth chamber under standard greenhouse conditions (16 h light and 8 h darkness at 22 °C). For the gene expression analysis, the seeds of *Arabidopsis* were sown on Murashige and Skoog (MS) medium containing 0.7% agar and after a 3-day stratification at 4 °C, the seeds were grown in a growth chamber for 7 days, after which the whole plants were sampled for RNA extraction.

### 4.2. Cloning of the CsCTR1 Open Reading Frame (ORF)

The total RNA was extracted from the mature leaves of Cucumber cultivar S52 using the TRIpure reagent (Aidlab, Beijing, China) according to the kit instructions. After determining the quality and concentration of RNAs by spectrophotometry for the ratio of OD260/OD280 and gel electrophoresis, the total RNA (1 μg) was used to synthesise the first strand of cDNA according to the manual of the ReverTra Ace^®^ qPCR RT Kit (TOYOBO, Shanghai, China).

A BLASTP search using the amino acid sequence of the *AtCTR1* gene from *Arabidopsis thaliana* (GenBank accession No. NP_850760) against the protein database within the Cucumber Genome Database [41,42] was conducted, and a tentative consensus sequence of csa011978 located on chromosome 6 was identified. The csa011978 sequence showed 67% amino acid identity with NP_850760. Based on the nucleotide sequence of csa011978, a pair of primers covering the cDNA of csa011978 was designed to obtain the cDNA sequence containing the ORF as follows: CsCTR1-Fw 5'-TCCCCCGGGCACCCCTTCAACAATGGCG-3' with an XmaI site (underlined) and CsCTR1-Re 5'-CGAGCTCGTCCCTTGTCCCCTCAC-3' with a SacI site (underlined). The restriction sites were added to ends of the primers for easy subcloning into the expression vector. The cDNA clone of the cucumber *CTR1* gene was amplified using cDNA from the mature leaves as a template using PrimeSTAR Max (TaKaRa, Shanghai, China) under following conditions: 30 cycles of 10 s at 95 °C, 15 s at 55 °C and 30 s at 72 °C, and a final 20 min at 72 °C (add 1.5 U Taq enzyme and 3 μL dNTP Mixture, TaKaRa, Shanghai, China). The PCR product was purified, ligated into the pMD18-T vector to form *pMD18-CsCTR1*, transferred into *E. coli* strain DH5α, and then sequenced. The resulting cucumber *CTR1* gene was designated *CsCTR1*. The sequence of *CsCTR1* has been deposited in GenBank under the accession number JQ277220.

### 4.3. Bioinformatic Analysis

GenScan [43] was used to predict the ORF. The gene structure analysis was conducted using GSDS [44]. The theoretical molecular weight (*M*w) was calculated using the ExPASy compute pI/*M*_W_ tool [45]. The similar sequences of other species were obtained using the BLASTP tool [46]. A multiple sequences alignment was performed using ClustalX 2.0 [47] and processed using GeneDoc [48]. MEGA (Molecular Evolutionary Genetics Analysis, version 2.1, The Biodesign Institute, Tempe, AZ, USA) was subsequently used to construct the phylogenetic tree using the neighbour-joining method.

### 4.4. Construction of Vector for Transformation

The transgenic construct (*pBI121-35s-CsCTR1*) was designed to constitutively overexpress a functional *CsCTR1* with the Cauliflower Mosaic Virus (CaMV) 35S promoter and employing the nopaline synthase (nos) 3' terminator. Firstly, the vector *pMD18-CsCTR1* and empty expression vector pBI121 were digested by *XmaI* and *SacI*, respectively. Then the *CsCTR1* fragment excised from *pMD18-CsCTR1* and the large fragment digestion product of pBI121 were ligated by T4 DNA ligase (Cwbiotech, Beijing, China). The constitutive-expression plasmid *pBI121-35s-CsCTR1* was transformed into *E. coli* strain DH5α by electroporation and the construct was verified by sequencing.

### 4.5. Construction of Vector for Transformation

The plasmid *pBI121-35s-CsCTR1* with kanamycin resistance was transformed into *Agrobacterium tumefaciens* strain GV3101 by electroporation. The seeds of the *Arabidopsis ctr1-1* mutant were sown onto Murashige and Skoog (MS) medium containing 0.7% agar. After a 3-day stratification at 4 °C, the seeds were grown in a growth chamber (16 h light and 8 h darkness at 22 °C). One week later, the plants were transplanted to soil, where they have continued to grow. The *A. tumefaciens*-mediated transformation of *Arabidopsis* was performed using the floral dip method [49]. The T1 seeds were collected and sown onto sterile half-strength MS medium containing 50 mg/L kanamycin. To select the transformants, the kanamycin-resistant seedlings were transferred to the soil, and the genomic DNA was extracted from each putative transformant. The positive transformants were identified by PCR using *CsCTR1*-specific primers. Semi-quantitative PCR and quantitative real-time PCR were performed to further investigate the expression level of the transformants. The plants with high expression levels were selected to continue a pure line of selection. The T2 generation was tested for kanamycin resistance, resulting in a segregation ratio of 3:1. In T3 generation the lines that showed 100% resistance to kanamycin were considered to be homozygous.

### 4.6. Seedling Triple-Response Assay

The *Arabidopsis ctr1-1* mutant, the Columbia-0 wild type (col) and the transformant seeds were surface-sterilised with 20% bleach for 10–15 min. Subsequently, the seeds were washed five times with distilled water and then sown on MS medium containing 0.7% agar and cold-treated for 3 days at 4 °C before germination and growth in the dark at 22 °C for 5 days in the dark [2]. A total of 15 plants in each group were used to measure the length of the hypocotyls and roots.

### 4.7. Quantitative Real-Time PCR

The qRT-PCR was performed according to Bie *et al.* (2013) [33]. A dissociation curve was performed to ensure the validity of each specific PCR product. The qRT-PCR was repeated three biological replicates using the cucumber *Actin3* gene or the *Arabidopsis Actin2* gene as an internal standard. The primers that were used in the qRT-PCR are listed as follows: *CsCTR1*, 5'-CTTCACCATTGCGATTTCC-3' (forward) and 5'-TCAGTTGGGCTGCTTTTTT-3' (reverse); *CsActin3*, 5'-TCGTGCTGGATTCTGGTG-3' (forward) and 5'-GGCAGTGGTGGTGAACAT-3' (reverse); *AtEBP*, 5'-CCAAGGCCAAGGGCCGTAAA-3' (forward) and 5'-TCACGTTAACTTGGTTGGTGGGA-3' (reverse); *AtERF1*, 5'-TCGAGCAGTCCACGCAACAA-3' (forward) and 5'-TCGAACGTCCCGAGCCAAAC-3' (reverse); *AtHCHIB*, 5'-CTTACAACGCCTTTATCACCGCT-3' (forward) and 5'-ATTGGTCCTCTTCCGTAGTAGCG-3' (reverse); and *AtActin2*, 5'-TTGTCACACACAAGTGCATCAT-3' (forward) and 5'-TTGTCACACACAAGTGCATCAT-3' (reverse).

### 4.8. Statistical Analyses

All of the statistical evaluations were performed using the SPSS (version 17, SPSS Inc., Chicago, IL, USA) software package. The results are presented as the mean ± SD (standard deviation) where applicable. A one-way ANOVA was used to determine the statistically significant differences. A significant difference was accepted at *p* < 0.05.

## 5. Conclusions

In summary, CTR1 is a key negative regulator of the ethylene signal transduction pathway, playing important roles in the regulation of plant development. We isolated and characterised the *CTR1* gene from cucumber by its ectopic expression in an *Arabidopsis ctr1-1* mutant and investigated its expression patterns in different plant organs and during different stages of floral developmental, as well as in response to external ethylene. This study provides a foundation for further investigations into the role of CTR1 protein factors in the relevant biological processes of cucumber and the molecular mechanism of the cucumber ethylene signaling pathway.

## Supplementary Materials

Supplementary Tables can be found at: http://www.mdpi.com/1422-0067/15/9/16331/s1.

## Supplementary Files

Supplementary File 1
