# Supplementary material for: Ectopic Expression of CsCTR1, a Cucumber CTR-Like Gene, Attenuates Constitutive Ethylene Signaling in an Arabidopsis ctr1-1 Mutant and Expression Pattern Analysis of CsCTR1 in Cucumber (Cucumis sativus)
Source: Int J Mol Sci. 2014 Sep 15;15(9):16331–50. doi: 10.3390/ijms150916331 (PMC4200800; doi:10.3390/ijms150916331)
Supplement: Supplementary File 1 [file ijms-15-16331-s001.pdf]

Supplementary Information

Table S1. Root length of the seedlings grown in the light.

Seedling Lines	Average of Root Length (mm)	Standard Deviation
col	11.525 ^{a}	0.127
ctr1-1	5.184 ^{d}	0.179
33	7.430 ^{c}	0.073
15	8.174 ^{bc}	0.093
47	8.465 ^{b}	0.046

The statistical significance was determined by Duncan's multiple comparison tests. Different letters above bars indicate significant differences ($p < 0.05$). The same letter indicates no significant difference ($p < 0.05$).

Table S2. Root and hypocotyl length of the etiolated seedlings.

Seedling Lines	Average of Root Length (mm)	Standard Deviation	Average of Hypocotyl Length (mm)	Standard Deviation
col	5.254 ^{a}	1.295	14.294 ^{a}	1.704
ctr1-1	1.011 ^{c}	0.220	7.735 ^{c}	0.629
33	2.641 ^{b}	0.378	8.952 ^{b}	0.439
15	2.740 ^{b}	0.600	9.216 ^{b}	0.375
47	2.698 ^{b}	0.439	9.216 ^{b}	0.493

The statistical significance was determined by Duncan's multiple comparison tests. Different letters above bars indicate significant differences ($p < 0.05$). The same letter indicates no significant difference ($p < 0.05$).
